# Probability of referral to curative dental treatment in a preventive pediatric dentistry program

**DOI:** 10.1590/1807-3107bor-2025.vol39.039

**Published:** 2025-04-04

**Authors:** Lorena Castro ROCHA, Cristiane Meira ASSUNÇÃO, Larissa de Moura SEVERINO, Cristiane Baccin BENDO, Lucas Guimarães ABREU, Sheyla Márcia AUAD

**Affiliations:** (a) Universidade Federal de Minas Gerais – UFMG School of Dentistry, Department of Pediatric Dentistry, Belo Horizonte, MG, Brazil.

**Keywords:** Preventive Dentistry, Pediatric Dentistry, Dental Care

## Abstract

Preventive maintenance in pediatric dentistry is essential for monitoring oral health and promoting healthy habits. This study aimed to evaluate how the frequency of follow-up appointments, as well as sex- and age-related differences, impact the probability of referral for curative treatment among pediatric dentistry patients at a Brazilian dental school. This retrospective longitudinal study included dental records of patients who had their first appointment between the second half of 2013 and the second half of 2019, completed treatment, and returned for at least one check-up visit. Descriptive and survival analyses were performed. A total of 296 dental records were evaluated, comprising 47.6% female and 52.4% male patients. The likelihood of referral to curative treatment increased with longer follow-up intervals, reaching 18.9% at six months and 82.7% at 48 months. Patients aged 9 years or younger were 2.07 times more likely to be referred to curative treatment than those aged 10 years or older. No significant difference was observed in referral probability between boys and girls. Longer intervals between check-up visits increased the likelihood of referral to curative treatment, which was higher among older children. Establishing personalized follow-up intervals based on individual patient needs is crucial for maintaining oral health.

## Introduction

The preventive check-up dental visit is a key instrument in pediatric dentistry for the longitudinal monitoring of children, allowing for the assessment of their growth, development, and overall health status. Follow-up visits contribute to the prevention and early detection of dental caries and other oral conditions, while also helping to reduce anxiety and fear of dental treatment, ultimately benefiting the physical well-being and academic performance of pediatric patients. Additionally, these visits facilitate a deeper understanding of the health-disease process and help demystify conventional curative dentistry.^1-3^

Dental caries is the most prevalent oral condition in pediatric dentistry, and its treatment is costly, negatively affecting the quality of life and daily activities of children and their families.^4,5^ This disease has a strong social component, with higher prevalence and severity observed in economically disadvantaged populations with lower educational levels.^6,7^ Therefore, assessing caries risk and determining appropriate intervals between visits are essential for preventing the development of new lesions and the progression of existing ones.^8^

Considering that biofilm accumulation plays a central role in the development of both caries and periodontal disease, assessing periodontal risk is an essential component of the routine examination of pediatric dentistry patients.^1^ The Visible Plaque Index (VPI) and Gingival Bleeding Index (GBI) can be used to evaluate biofilm control. Patients with high biofilm levels have an increased risk of restoration failure and, consequently, a greater need for curative visits.^9^

The greatest challenge for dentists is determining the ideal recall period, as this interval is still not supported by strong scientific evidence,^10^ despite being debated for over forty years.^11^ The evidence for establishing a standardized recall interval protocol to reduce the incidence of dental caries remains weak.^12^ A recent study conducted in Pakistan determined risk-based recall intervals for dental caries among 11- to 12-year-olds using the International Caries Detection and Assessment System (ICDAS) lesion classification. The intervals varied depending on whether the lesions were cavitated or non-cavitated, ranging from 6 months for high-risk children to up to 18 months for those at medium or low risk.^13^ It is important to note that the use of ICDAS should be complemented by an assessment of lesion activity to improve risk classification and establish appropriate recall intervals.

Ensuring that patients return to dental appointments should be a continuous process.^14^ Raising awareness among parents and guardians about the importance of preventive follow-up visits also remains a challenge.^15^ Current studies indicate that recall intervals for dental appointments should be individualized based on patient risk assessment, response to treatment, and history of oral diseases.^1,3,12,14,16,17^

The School of Dentistry at the Federal University of Minas Gerais (FAO-UFMG), located in Belo Horizonte, southeastern Brazil, runs a preventive dental maintenance program for children and adolescents treated at the pediatric dentistry clinic, where patients are periodically recalled for evaluation. During these visits, preventive procedures are performed, and the need for referral to curative treatment is assessed. A previous study conducted at the same institution with 127 patients found that 64.3% were recalled for follow-up appointments once a year, while 34.8% were recalled twice a year.^18^ However, this study was conducted two decades ago, and significant changes in clinical practices, preventive strategies, and patient behaviors may have occurred since then. Therefore, the present study aimed to provide a more updated and comprehensive understanding of patient care in this context.

Considering the importance of preventive follow-up appointments for adequate oral health monitoring, this study aimed to evaluate the impact of follow-up visit frequency, as well as sex- and age-related differences, on the probability of referral for curative treatment among patients at the pediatric dentistry clinic, FAO-UFMG. The hypothesis considered was that longer intervals between preventive appointments would increase the likelihood of referral for curative treatment.

## Methods

### Study design and population

This retrospective, longitudinal, census-type study was conducted at the Department of Pediatric Dentistry (SCA), School of Dentistry, Federal University of Minas Gerais (FAO-UFMG). UFMG is a public university, and most of the patients treated at the School of Dentistry are from the national public health system and/or economically disadvantaged backgrounds. Pediatric dental care is provided by undergraduate students under the supervision of faculty advisors, at no cost to the patients.

Undergraduate students take compulsory courses with different characteristics. In the “Individual Prevention of Caries and Occlusion Problems” (Prevention) course, follow-up visits are conducted, during which clinical examination, Visible Plaque Index (VPI) and Gingival Bleeding Index (GBI) assessment, biofilm control, fluoride application (when indicated), and dietary guidance are performed. Curative appointments are conducted in the “Comprehensive Care for the Child” (Attention) course, which involves microinvasive, invasive, endodontic, and surgical treatments; preventive treatment is also provided, complementing the curative care.

The “Prevention” course accommodates patients who have completed curative treatment and were discharged from the clinical activities of the “Attention” course, as well as patients who do not require curative treatment. Patients are called for periodic visits to promote the maintenance of oral health, address emerging treatment needs early, and empower patients and their families as active participants in the health-disease process through individualized and collective oral health guidance.

Patients requiring curative treatment during follow-up visits are referred to the “Attention” course. Thus, based on the classification of caries disease activity and the need for curative or preventive treatment, patients are referred between courses.

### Sample and eligibility criteria

Data was collected from the dental records of patients treated at the pediatric dentistry clinic. A total of 1,390 dental records of patients who had their first appointments and completed treatment between the second half of 2013 and the second half of 2019 were eligible. This period marks the beginning of activities in the “Prevention” course and includes records of follow-up appointments up to the last semester before the temporary interruption of services due to the COVID-19 pandemic.

The records were included in the study if they met the following criteria: (a) dental records of patients who had their first appointment in one of the mandatory SCA courses starting from the second half of 2013; (b) dental records of patients who were discharged at least once after the first appointment and attended a preventive maintenance visit; and (c) dental records which included a signed informed consent form provided by the patient’s parent or guardian. Incomplete dental records, those with missing pages, or records of patients with specific treatment needs, such as patients with special needs not covered by the SCA courses and referred for clinical care in other dental school sectors, were excluded.

### Ethical considerations

This project was approved by the Human Research Ethics Committee, Federal University of Minas Gerais (CEP/UFMG) (CAAE: 44625221.4.0000.5149). It is important to note that an informed consent form was included in all dental records, in which the patient’s parents or guardians consented to the use of the information collected for scientific research.

### Calibration and pilot study

Calibration of the researcher responsible for data collection was conducted in two stages under the guidance of a gold-standard researcher. The first stage involved theoretical training on the aspects to be evaluated in the dental records. Subsequently, 15 dental records were analyzed by both the researcher (LCR) and the gold-standard researcher (SMA) to determine inter-examiner agreement (inter-examiner Kappa: 0.881). The researcher then re-evaluated the same dental records after two weeks to assess intra-examiner agreement (intra-examiner Kappa: 0.888). The calibration process also served as a pilot study to test the methodology, and no changes were required for data collection. The dental records evaluated in the pilot study were not included in the main study.

### Data collection

To assess the outcome of referrals for dental treatment between follow-up appointments, an analysis of patients’ pathways between the two courses was conducted. The study variable was the number of preventive or curative visits. Preventive maintenance events or referrals to curative appointments were recorded based on the patient’s first and follow-up visits, both occurring between the second half of 2013 and the second half of 2019. The patient pathway form included the following: (a) the interval between follow-up appointments for patients without treatment needs; (b) the interval between the completion of curative treatment and the follow-up appointment for patients with treatment needs; and (c) the patients’ status at the end of each follow-up visit (whether they were discharged or required additional treatment).

### Statistical analysis

Data were stored and descriptive analyses were performed using SPSS for Windows software (IBM SPSS Statistics for Windows, Version 21.0., Armonk, USA: IBM Corp.). The evaluation of patients’ pathways between maintenance and curative appointments was conducted through survival analysis, using MedCalc software (MedCalc Software bvba, Ostend, Flanders, Belgium). For the survival analysis, the time interval between visits (in months), the type of visit (preventive or curative), and patient gender and age at the time of the visit were considered.

The Kaplan-Meier test was used to calculate the probability of referral to treatment for patients from the pediatric dentistry clinic at FAO-UFMG. The analysis unit was the number of dental appointments of patients being followed up at the clinic, rather than the number of patients.

Cox analysis was used to compare the likelihood of referral for treatment between female and male patients, as well as between children (≤ 9 years) and adolescents (≥ 10 years). The classification of patients aged ≤ 9 as children and those aged ≥ 10 as adolescents was based on the World Health Organization manual (WHO, 2006). The results of the Cox analysis were presented as hazard ratios (HR) with 95% confidence intervals (CI).

## Results

A total of 296 dental records were analyzed, of which 141 were from female patients (47.6%) and 155 were from male patients (52.4%). The mean age of patients at the first visit was 5.97 years (SD = 2.305).

**Figure 1:**
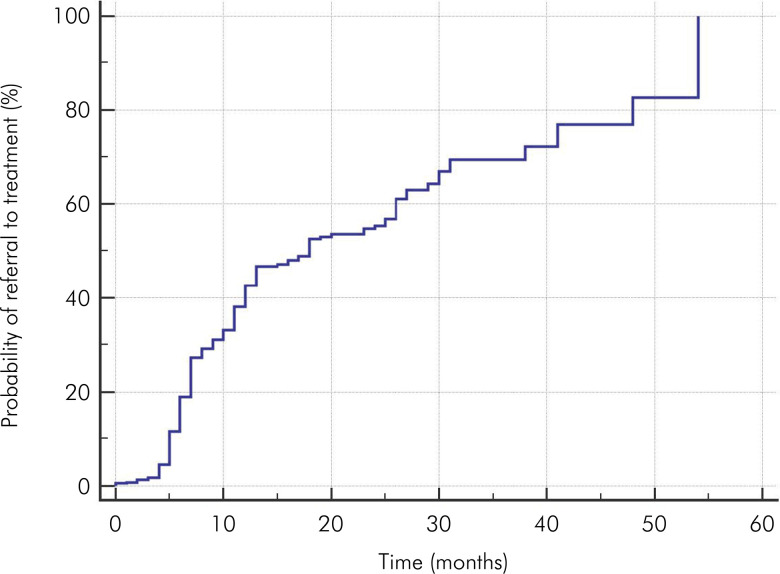
Probability curve illustrating the likelihood of referral to curative treatment over time (in months).

Data from 972 dental appointments were aggregated for the referral-to-treatment outcome. Among these, 281 (28.9%) required curative treatment, whereas 691 (71.1%) did not. The mean follow-up time was 24.32 months (standard error = 1.03). The probability of needing a referral for curative treatment at six months of follow-up was 18.9%. At the 48-month follow-up, the likelihood of requiring a referral for curative treatment increased markedly to 82.7% (Table). Figure 1 shows the probability curve for the need for referral for treatment over time, indicating that longer intervals between appointments were associated with a higher likelihood of referral for curative treatment.

**Table:** Probability of referral to curative treatment among patients at the pediatric dentistry clinic, School of Dentistry, Federal University of Minas Gerais (FAO-UFMG).

Time (months)	# cases	Patient situation		Accumulated events	Remaining events	Probability of referral to curative treatment (%)
		Referred to curative treatment	Kept in preventive maintenance			
-	-	-	-	-	972	-
3	113	16	97	16	859	1.7
6	444	126	318	142	415	18.9
9	178	57	121	199	237	31.1
12	97	35	62	234	140	42.6
15	21	11	10	245	119	47.1
18	30	11	19	256	89	52.5
21	9	2	7	258	80	53.6
24	15	3	12	261	65	55.4
27	33	10	23	271	32	63
30	7	3	4	274	25	66.9
34	13	2	11	276	12	69.5
36	1	0	1	276	11	69.5
39	5	1	3	277	6	72.3
42	2	1	1	278	4	76.9
48	2	1	1	279	2	82.7
54	2	2	0	281	0	100

**Figure 2:**
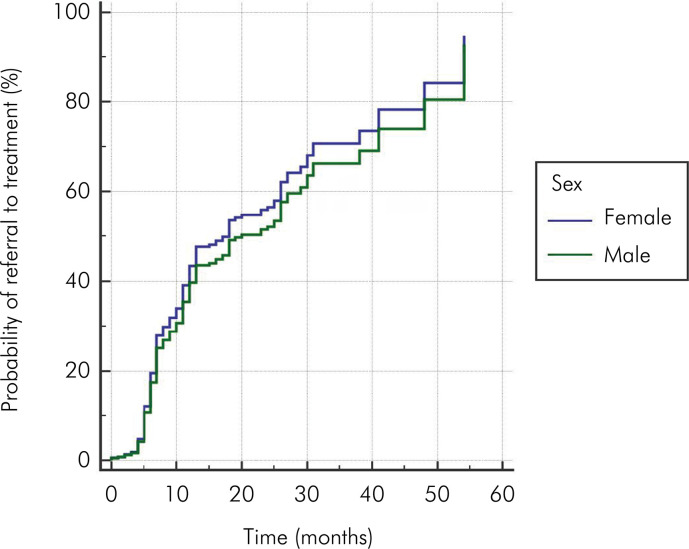
Probability curve illustrating the likelihood of referral to curative treatment over time (in months), stratified by the patient’s sex.

**Figure 3:**
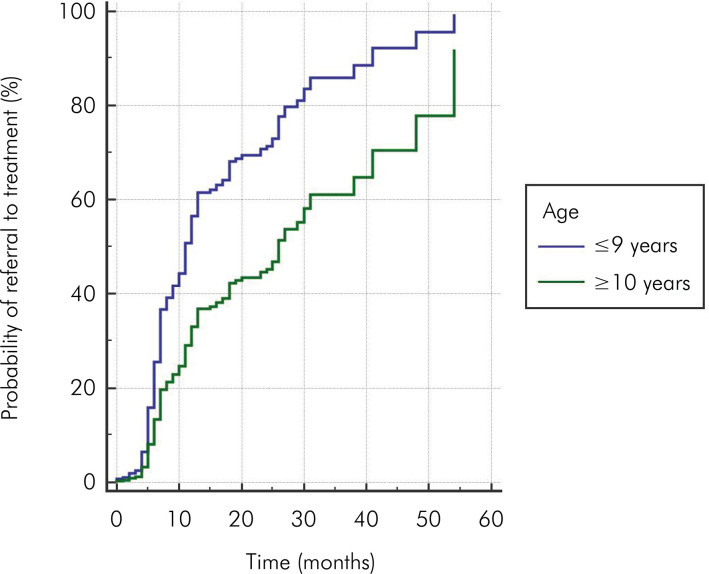
Probability curve illustrating the likelihood of referral to curative treatment over time (in months), stratified by age group (< 9 years and ≥ 10 years).

Cox analysis showed no difference between the results for female and male patients regarding the probability of referral to curative treatment during the evaluated follow-up period (HR = 1.13; [0.89– .43]; p = 0.304) (Figure 2). Patients aged 9 years or younger were 2.07 times more likely to be referred to curative treatment over the follow-up period than patients aged 10 years or older (HR = 2.07; [1.62–2.64]; p < 0.001) (Figure 3).

## Discussion

Longitudinal dental care, particularly during childhood, is crucial for establishing and maintaining healthy habits. However, the logistics of providing this continuous care present a challenge, especially for services linked to educational institutions.

The preventive maintenance program at FAO-UFMG is structured around biannual patient care, representing an innovative initiative within a public higher education institution. This program serves children from diverse regions and socioeconomic backgrounds, ensuring longitudinal monitoring and follow-up of their oral health. By evaluating the program’s efficiency and tracking its outcomes over time, it is possible to assess its impact, identify areas for improvement, and provide valuable insights into the implementation of similar initiatives in other contexts, particularly in public health settings. These findings underscore the program’s significance in promoting equitable access to preventive oral healthcare and its potential as a model for broader clinical applications.

However, this biannual periodicity is not always followed, either due to the need for more frequent follow-up visits or patient absences. As a result, the time between follow-up visits varied widely (from 3 to 54 months), with a mean follow-up time of approximately 24 months. The longer recall period can be explained by the temporary interruption of clinical care due to the COVID-19 pandemic, as the activities of the mandatory disciplines were suspended in the first half of 2020 and resumed only in the second half of 2021. Of the 296 dental records, 68 belonged to patients whose first visits occurred before the second half of 2019, and these patients were gradually recalled starting in 2021.

In the present study, a recall interval of 6 months between preventive maintenance appointments was maintained, demonstrating that the primary objective of the program—semiannual patient recall—was achieved. A longitudinal study conducted in Norway evaluated the duration of recall intervals for dental examinations in children aged 5 to 12 and investigated the association between the time interval between appointments and the prevalence of caries. The intervals ranged from 4 to 30 months, with 12, 18, 20, and 24 months being the most common. The analyses revealed that the probability of having a shorter interval between visits was higher in children with caries compared to those without. The study concluded that the intervals between appointments were individualized and extended, suggesting that more resources were allocated to children with a greater need for dental treatment, thereby reducing oral health inequality^17^. It is important to note that, at the FAO-UFMG pediatric dentistry clinic, dental treatment is provided free of charge to patients, most of whom come from less privileged socioeconomic backgrounds and have a history of caries, justifying the adoption of semiannual follow-up visits.

There was no difference in the probability of referral to curative dental treatment between female and male patients. Regarding the sex of the child, the literature remains inconclusive, with studies reporting mixed results about whether boys or girls are at higher or lower risk for developing new carious lesions.^6,7,19^ The absence of association with sex in this study may be attributed to factors unrelated to biological differences, such as similar levels of exposure to oral health education, oral hygiene habits, preventive care, or dietary habits among boys and girls within the analyzed population. In contrast, different results were observed by Abanto et al.,^8^ who found a significant association between male children and the inactivation of carious lesions at the time of the follow-up visit, suggesting that behavioral or access-related factors might mediate outcomes in different contexts. Further investigation is needed to clarify whether sociocultural, behavioral, or healthcare access disparities contribute to sex-related differences in caries management and treatment referral patterns.

In the present study, patients aged 9 or less were 2.07 times more likely to be referred to curative treatment than patients aged 10 or more. Our study did not investigate the reason for referral to treatment; however, considering data from Brazilian studies conducted with a population with a similar socioeconomic profile^9,20^, we suggest that patients aged 9 or less were more likely to be referred to curative dental treatment in follow-up visits due to restorative failure. This hypothesis is reinforced by the results of Chisini et al.,^21^ who observed that restorative failures may be associated with the patients’ behavior towards dental care, due to a greater difficulty in collaboration by younger children.^21^

The present study observed that shorter follow-up intervals led to less need for referral to curative treatment. This is shown in Figure 1, where the curve illustrating the probability of needing referral to curative treatment over time increases as the time between follow-up appointments lengthens. A non-randomized controlled longitudinal study observed that a two-to-three-month follow-up interval, associated with oral hygiene instructions, significantly reduced the occurrence of dental caries and the progression of periodontal disease.^22^

The probability of requiring a referral for curative treatment after six months of follow-up was 18.9%, rising to 31.1% after 9 months and 42.6% after 12 months. This suggests that the longer the interval between follow-up visits, the greater the likelihood of patients displaying compromised oral health. Similar results were observed by Loken et al.,^17^ who recorded shorter intervals for children with caries experience and the need for curative treatment, compared to children who had not experienced carious lesions.

Dental prevention programs, including the preventive follow-up program of pediatric dentistry at UFMG, provide careful monitoring of patients’ oral health.^18^ In a study evaluating a municipal dental prevention program in the city of São Paulo, Brazil, a significant reduction in the biofilm index, gingival bleeding index, and initial caries lesions was observed between the initial and follow-up visits of children who attended three follow-up appointments. For each follow-up visit, there was a 77% reduction in the risk of new early carious lesions and a significantly increased likelihood of remineralization of early active carious lesions.^8^

A systematic review presented little evidence for a single recall interval protocol for all patients to reduce the incidence of dental caries.^3^ As evidenced by other studies, individualized follow-up intervals should be based on each patient’s risk assessment.^1,14,17^ The risk of caries can be determined by evaluating individual factors through a comprehensive assessment that considers the balance between disease indicators (clinical observations), biological risk factors, such as diet and oral hygiene habits, and protective factors, like fluoride exposure.^23,24^ This holistic approach enables dental professionals to develop personalized preventive and treatment plans tailored to each patient’s specific needs, enhancing the effectiveness of caries management strategies.

In personalized recall intervals, resources are saved by extending the intervals for children at low risk of caries and directing resources to patients at high disease risk, thus contributing to diminishing inequalities in oral health programs.^17^ This targeted approach not only improves the efficiency of care but also plays a significant role in reducing oral health disparities by prioritizing the needs of vulnerable populations.

For dental professionals, adopting individualized risk-based recall intervals represents a practical, economical, efficient, and impactful strategy in routine practice. It enhances patient care by ensuring timely interventions for high-risk individuals while avoiding unnecessary visits for low-risk patients, thereby reducing caregiver burden and promoting adherence to preventive care. In teaching institutions, such as the pediatric dentistry program at FAO-UFMG, implementing this model could optimize resource utilization, improve student training in risk-based management, and serve as a reference for public health programs. Ultimately, this approach underscores the importance of personalized care in achieving sustainable and equitable oral health outcomes.

Sheiham et al.,^25^ evaluated differences in oral status between “regular” and “irregular” patients (those who visited a dentist only when there was a demand). Irregular patients had a higher number of decayed and missing teeth when compared to regular patients. Although regular participants had fewer active carious lesions, the number of teeth restored among them was higher,^25^ which suggests that a shorter recall interval may be associated with overtreatment. The present study did not assess this issue, but it is a point to be studied in future research, especially with the recent incorporation of the minimal intervention philosophy, which can positively contribute to a lower rate of overtreatment and greater attention to non-invasive and preventive procedures.

Dental caries risk assessment should be performed at the patient’s first visit and updated during preventive follow-up visits.^26^ The dental professional may encounter barriers to engaging parents in preventive dental care, as evidenced by a systematic review.^27^ Addressing these challenges with personalized approaches and tailored communication strategies can help overcome obstacles and increase parental involvement in their child’s oral health^28^. Dental professionals must consider each child’s unique characteristics to implement appropriate recall strategies, preventive care, and targeted interventions based on the identified risk and caries activity. By doing so, they can provide more effective, individualized care that supports both the child’s health and the parent’s role in maintaining it.

This study has limitations. Considering the incomplete information in the dental records, it was not possible to assess, as initially planned, the contribution of oral hygiene standards, represented by the gingival bleeding (GBI) and visible plaque (VPI) indices, to the referral for curative dental treatment. The information contained in the dental records also did not make it possible to identify the demands for curative treatment. This is an interesting point to be investigated in future research, which may include clinical data such as an increase in caries lesions, biofilm control levels, and sugar consumption patterns. The results may not be fully generalizable to other populations, as they were derived from a specific population, and the retrospective nature of the study presents inherent limitations, including potential biases related to case selection and the accuracy of the recorded data. These factors should be considered when evaluating the outcomes and their implications.

## Conclusion

The present results showed that the longer the interval between recall visits, the greater the likelihood of referral to curative treatment, i.e., the higher the risk of oral health deterioration among patients enrolled in the preventive maintenance program at FAO-UFMG. This finding underscores the importance of periodically assessing patients’ caries risk to establish individualized follow-up intervals, thereby ensuring closer attention to those with greater needs while optimizing resource allocation.
